# Supplementary material for: Esports experts have a wide gaze distribution and short gaze fixation duration: A focus on League of Legends players
Source: PLoS One. 2024 Jan 2;19(1):e0288770. doi: 10.1371/journal.pone.0288770 (PMC10760684; doi:10.1371/journal.pone.0288770)
Supplement: S1 File — (DOCX) [file pone.0288770.s001.docx]

***PLoS One* Supporting Information file S1**Article title: Esports experts have a wide gaze distribution and short gaze fixation duration: A focus on League of Legends players

Authors: Inhyeok Jeong, Kazutoshi Kudo, Kimitaka Nakazawa


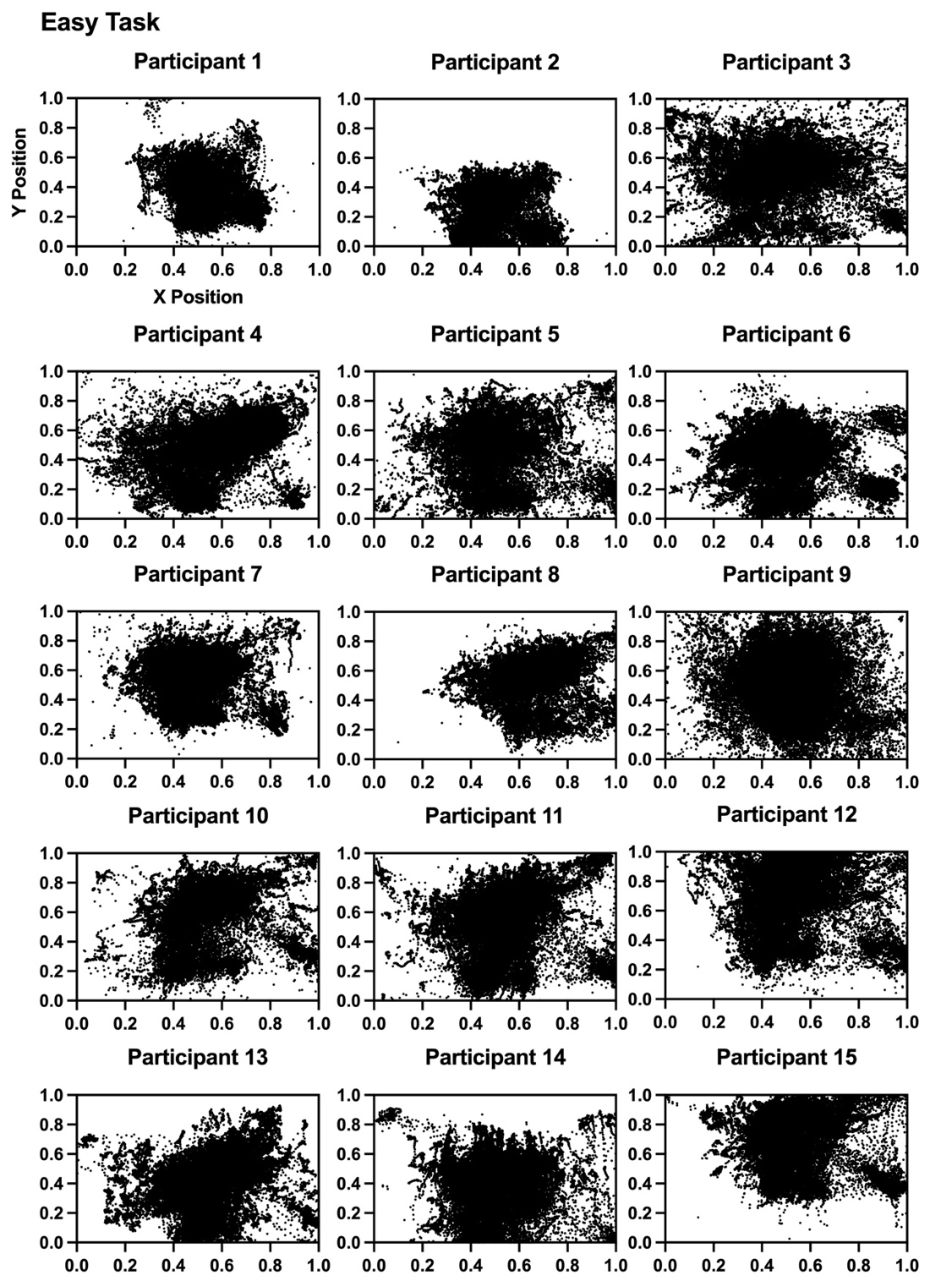


**Supplementary Figure 1.** Total gaze movement of each participant (Easy Task, Participants 1 to 15).


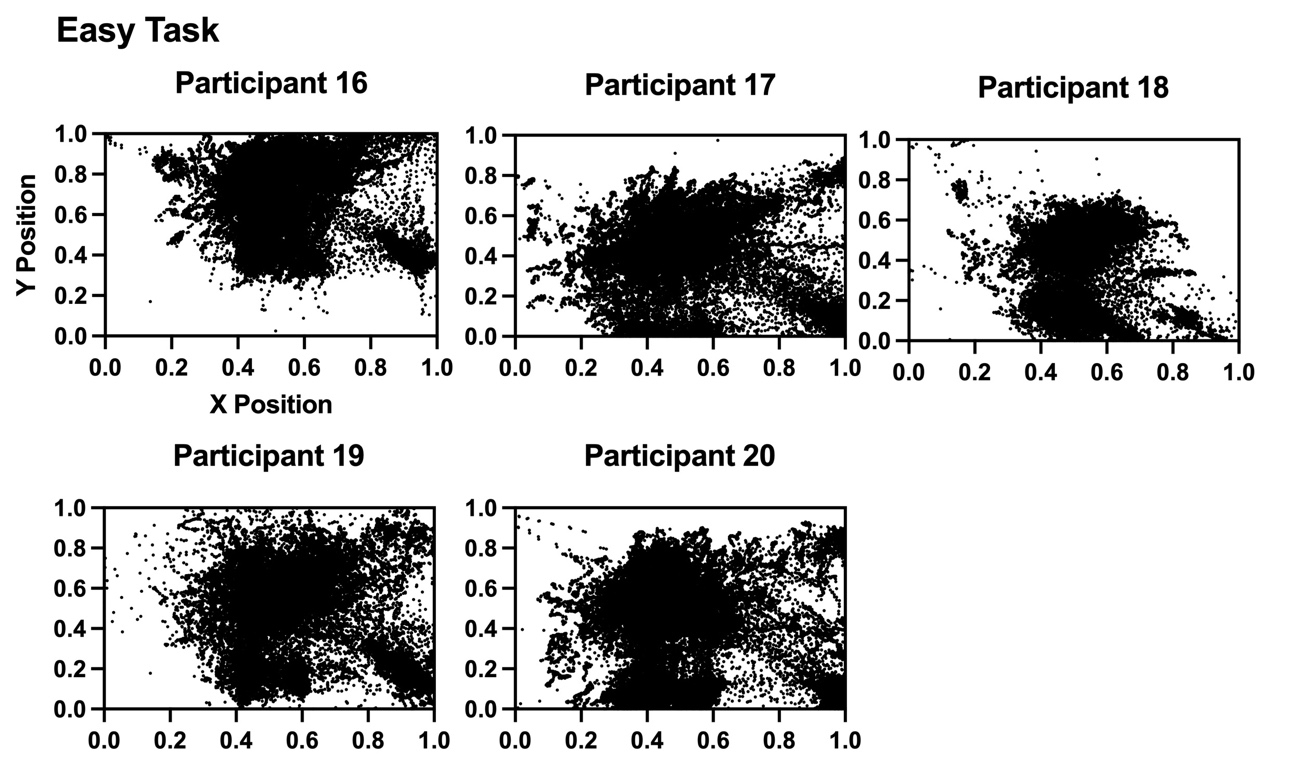


**Supplementary Figure 2.** Total gaze movement of each participant (Easy Task, Participants 16 to 20).

**
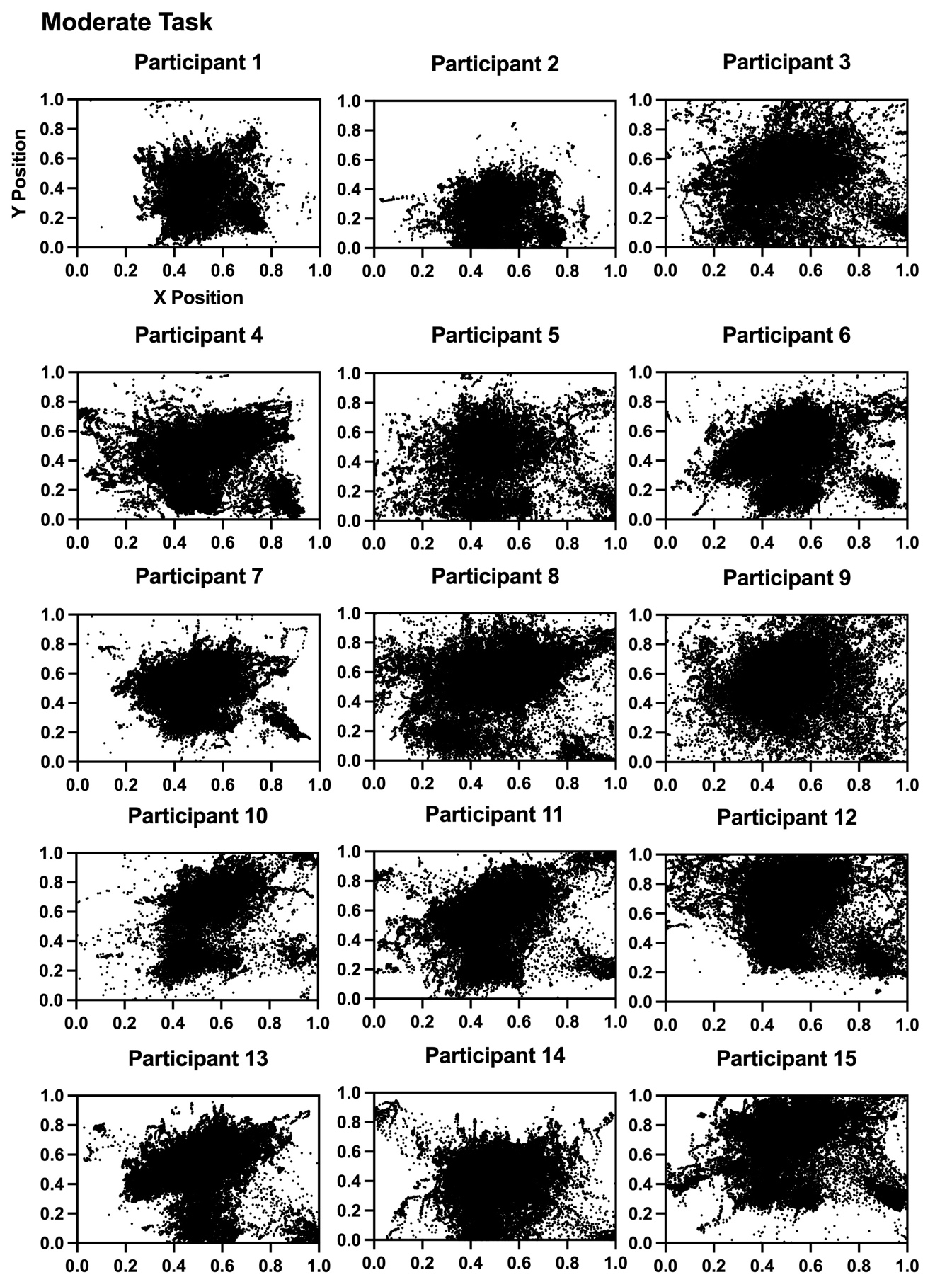
**

**Supplementary Figure 3.** Total gaze movement of each participant (Moderate Task, Participants 1 to 15).

**
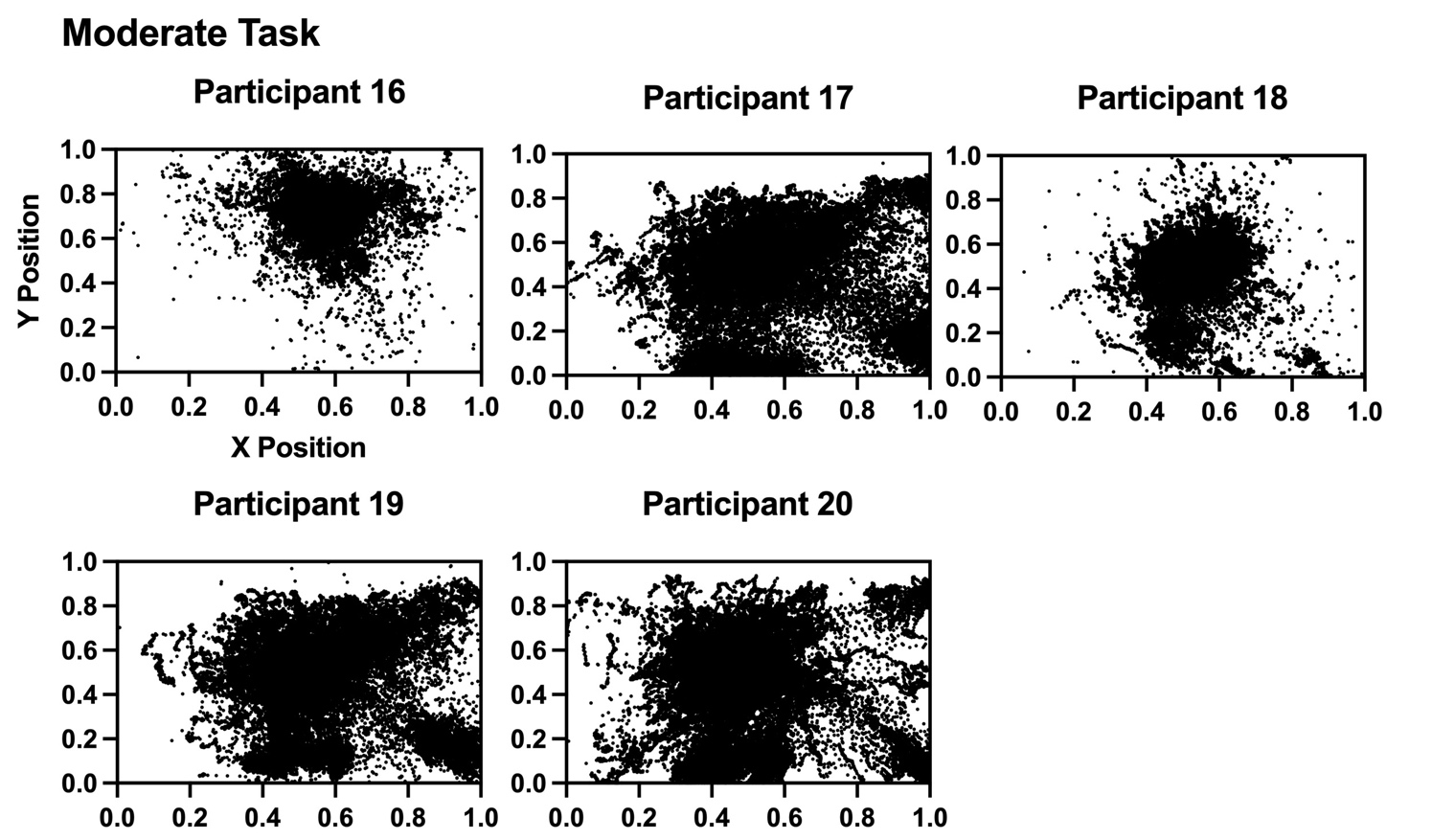
**

**Supplementary Figure 4.** Total gaze movement of each participant (Moderate Task, Participants 16 to 20).
