# Supplementary material for: Esports experts have a wide gaze distribution and short gaze fixation duration: A focus on League of Legends players
Source: PLoS One. 2024 Jan 2;19(1):e0288770. doi: 10.1371/journal.pone.0288770 (PMC10760684; doi:10.1371/journal.pone.0288770)
Supplement: S2 File — (DOCX) [file pone.0288770.s002.docx]

***PLoS One* Supporting Information file S**Article title: Esports experts have a wide gaze distribution and short gaze fixation duration: A focus on League of Legends players

Authors: Inhyeok Jeong, Kazutoshi Kudo, Kimitaka Nakazawa

**Raw data of each participant:** Expert (4, 1, 5, 0), Low Skill (29, 8, 32, 5)

**Power calculation for unpaired t-test between Expert (n = 4) vs. Low Skill (n = 4)**

**t test:** Means: Difference between two independent means (two groups)

**Analysis:** Compute required sample size

**Input:** Tail(s) = Two

Effect size d = 1.4

α err prob = 0.05

Power (1-β err prob)) = 0.8

Allocation ration N2/N1 = 1

**Output:** Noncentrality parameter δ = 3.13

Critical t = 2.10

Df = 18

Sample size group 1 = 10

Sample size group 2 = 10

Total sample size = 20

Actual power = 0.841
